# Mutation-driven epigenetic alterations as a defining hallmark of central cartilaginous tumours, giant cell tumour of bone and chondroblastoma

**DOI:** 10.1007/s00428-019-02699-2

**Published:** 2019-11-14

**Authors:** Sanne Venneker, Karoly Szuhai, Pancras C. W. Hogendoorn, Judith V. M. G. Bovée

**Affiliations:** 1 Department of Pathology, Leiden University Medical Center, Leiden, The Netherlands; 2 Department of Cell and Chemical Biology, Leiden University Medical Center, Leiden, The Netherlands

**Keywords:** Bone neoplasm, Chondrosarcoma, Giant cell tumour of bone, Chondroblastoma, *IDH* mutations, Histone H3.3 variants

## Abstract

Recently, specific driver mutations were identified in chondroblastoma, giant cell tumour of bone and central cartilaginous tumours (specifically enchondroma and central chondrosarcoma), sharing the ability to induce genome-wide epigenetic alterations. In chondroblastoma and giant cell tumour of bone, the neoplastic mononuclear stromal-like cells frequently harbour specific point mutations in the genes encoding for histone H3.3 (*H3F3A* and *H3F3B*). The identification of these driver mutations has led to development of novel diagnostic tools to distinguish between chondroblastoma, giant cell tumour of bone and other giant cell containing tumours. From a biological perspective, these mutations induce several global and local alterations of the histone modification marks. Similar observations are made for central cartilaginous tumours, which frequently harbour specific point mutations in the metabolic enzymes *IDH1* or *IDH2*. Besides an altered methylation pattern on histones, *IDH* mutations also induce a global DNA hypermethylation phenotype. In all of these tumour types, the mutation-driven epigenetic alterations lead to a highly altered transcriptome, resulting for instance in alterations in differentiation. These genomic alterations have diagnostic impact. Further research is needed to identify the genes and signalling pathways that are affected by the epigenetic alterations, which will hopefully lead to a better understanding of the biological mechanism underlying tumourigenesis.

## Introduction

The genetic make-up of tumour cells alone is insufficient to explain differences in cellular behaviour. One factor that can explain these differences is epigenetics: the stable and heritable change of gene function caused by other factors than alterations in the DNA sequence [1]. This involves mainly changes in the three-dimensional structure of DNA, which is defined by histones, nucleosomes and chromatin condensation. By altering the DNA structure, the accessibility for proteins involved in gene transcription is either enhanced or reduced, regulating gene expression.

To control DNA accessibility, several enzymes such as DNA methyltransferases, histone acetyltransferases, ubiquitin ligases and histone methyltransferases make modifications (e.g. methylation, acetylation, phosphorylation and ubiquitination) on DNA itself or on certain amino acid positions on histone tails [2]. At another level, chromatin remodelling complexes (e.g. SWI/SNF and INO80) construct, reposition or evict nucleosomes to change the packaging of the DNA [2]. Together, the dynamic and reversible epigenetic modifications define which genetic information is available for a cell and thereby regulate cellular fate and homeostasis.

Recently, it was shown that epigenetic regulatory genes are frequently mutated across several tumour types, leading to deregulation of normal gene expression patterns (e.g. silencing of tumour suppressor genes and activation of oncogenes) and thereby promotion of tumourigenesis [3]. Epigenetic alterations, unlike genetic causes of diseases, are reversible, making them interesting targets to develop novel anti-cancer therapies. In the past couple of years, several drugs targeting DNA methylation (i.e. azacitidine and decitabine) and histone acetylation (i.e. vorinostat, romidepsin and panobinostat) have been FDA approved for different haematological malignancies. Many clinical trials are ongoing to evaluate the effect of epigenetic drugs in a wide variety of tumour types, including advanced and metastatic sarcoma [4].

Bone and soft tissue tumours are a rare, heterogeneous group of mesenchymal tumours which frequently harbour epigenetic alterations. For instance, the promoter of the tumour suppressor gene PTEN is frequently hypermethylated in soft tissue sarcomas, while loss-of-function mutations in PTEN are rare in these tumours [5]. Furthermore, several bone and soft tissue tumours harbour an aberrant DNA methylation pattern across the whole genome (e.g. chondrosarcoma [6], Ewing sarcoma [7] and rhabdomyosarcoma [8]).

Deregulation of chromatin remodelling complexes is also commonly seen in sarcomas. For instance, loss of *SMARCB1* is the hallmark of malignant rhabdoid tumours and epithelioid sarcomas [9, 10]. *SMARCB1* is a core subunit of the SWI/SNF chromatin remodelling complex: a group of proteins involved in positioning the nucleosomes on the DNA. Furthermore, approximately 80% of all malignant peripheral nerve sheath tumours have mutations in the *EED* or *SUZ12* subunits of the polycomb repressive complex (PRC) 2 [11]. This complex is primarily involved in maintaining the repressive tri-methylation mark on lysine 27 of histone H3 (H3K27me3) which has led to the use of an easily applicable immunohistochemical diagnostic tool [12–14]. Moreover, certain translocations, such as the SS*18-SSX* fusion in synovial sarcomas, affect epigenetics. The *SS18* gene is involved in the SWI/SNF complex, while *SSX1* and *SSX2* are subunits of the PRC complexes [15]. Fusion of these genes leads to the formation of an altered chromatin remodelling complex which lacks the *SMARCB1* subunit, resulting in transcriptional repression of tumour suppressor genes (e.g. *EGR1*) and transcriptional activation of oncogenes (e.g. *SOX2*) [15, 16].

Mesenchymal tumours can also harbour mutations in histones themselves or genes indirectly related to epigenetic regulation. This review will focus on a group of bone tumours that share mutations in genes involved in epigenetic regulation: *H3 histone family member 3A and 3B (H3F3A and H3F3B)* mutations in giant cell tumour of bone and chondroblastoma, respectively, and *isocitrate dehydrogenase 1 and 2 (IDH1 and IDH2)* mutations in central cartilaginous tumours.

## Histone H3.3 variants in giant cell tumour of bone and chondroblastoma

### Giant cell tumour of bone

Giant cell tumour of bone (GCTB) is a locally aggressive and rarely metastasizing neoplasm (Table 1). These tumours typically arise in the end of long bones and are predominantly formed in skeletally mature young adults between the age of 20 and 45 [17]. Although GCTB has a high recurrence rate (~ 25% of patients), malignant transformation is very rare and occurs in less than 1% of the patients [32]. Pulmonary metastases are very rare and typically slow-growing. These are thought to represent pulmonary implants that result from embolization of intravascular growths of GCTB [33].

**Table 1:** Clinical and pathological characteristics of giant cell tumour of bone, chondroblastoma and central cartilaginous tumours

	Giant Cell Tumour of Bone [17]	Chondroblastoma [18]	Enchondroma [19]	Chondrosarcoma [20]
WHO classification	Intermediate (locally aggressive, rarely metastasizing)	Benign	Benign	Intermediate (locally aggressive): ACT Malignant: Grades I–III
Frequency	4–5% of all bone tumours	< 1% of all bone tumours	3–10% of all bone tumours	20% of all malignant bone tumours
Age	20–45 years	Typically between 10 and 25 years	5–80 years	> 50 years
Preferential location	Epiphysis of long bones	Epiphysis of long bones	Short and long tubular bones	Pelvis and meta-/diaphysis of long bones
Precursor lesion	–	–	–	None or enchondroma
Occurrence within syndrome	–	–	Maffucci syndrome and Ollier disease	Maffucci syndrome and Ollier disease
Histological grading	–	–	–	ACT and Grades I–III
Treatment	Curettage + bone grafting, en bloc resection, denosumab	Curettage + bone grafting, Radiofrequency ablation	Not treated	Curettage + bone grafting (ACT/Grade I), en bloc resection (Grade II/III)
Metastases	2%, pulmonary	Rare, ‘benign’ pulmonary	–	0% (ACT/Grade I), 10% (Grade II), 71% (Grade III) [21]
Recurrence	15–50%	14–18%	–	6–35% [22]
10-year survival rate	–	–	–	88% (ACT/Grade I), 62% (Grade II), 26% (Grade III) [23]
Radiology	Expansile, eccentric, often lobulated area of osteolysis, little matrix calcification	Lytic lesion, centric or eccentric, sclerotic border	Lytic lesion, mostly centric, well-marginated, radiolucent to heavily mineralised (ring and arc patterns)	Fusiform expansion, cortical thickening/erosion/destruction, radiolucent with mineralisation, ‘popcorn-like’ calcifications
Histomorphology	Mix of large multinucleated giant cells and round or spindle-shaped mononuclear cells	Chondroblasts with round to ovoid nuclei, osteoclast-like giant cells, chondroid matrix, ‘chicken-wire’ calcifications	Hypocellular, abundance of hyaline cartilaginous matrix, calcification	Increased cellularity, lobular, myxoid matrix changes, entrapment of pre-existing host lamellar bone
Recurrent mutations	*H3F3A* (G34) [24]	*H3F3A* and *H3F3B* (K36M) [24]	*IDH1* (R132) and *IDH2* (R172) [6, 25, 26]	*IDH1* (R132) and *IDH2* (R172), *COL2A1*, *TP53, YEATS2*, *NRAS* and IHH/PTHrP, pRB and PI3K/mTOR pathways [6, 25–29]
Immunohistochemistry	H3F3A G34W [30]	H3K36M [31], S100, DOG1	IDH1 R132H (low sensitivity) [6, 26]	IDH1 R132H (low sensitivity) [6, 26]

GCTB is histologically characterized by three types of cells: the multinucleated osteoclast-like giant cells, the mononuclear macrophage-like osteoclast precursor cells and the mononuclear spindle-shaped stromal cells. The latter are considered as the neoplastic component of GCTB; these cells have the ability to form tumours in mice and can be maintained in vitro [34, 35]. The neoplastic stromal cells are of osteoblastic origin and secrete high levels of chemokines (e.g. MCP-1 (CCL2) and SDF-1 (CXCL12)) to attract the mononuclear osteoclast precursor cells to the tumour site [36]. Subsequently, M-CSF (CSF1) secreted by the neoplastic stromal cells induces the expression of the RANK (TNFRSF11A) receptor on the attracted monocytes [37]. RANK ligand (TNFSF11) expression is upregulated by the neoplastic stromal cells, resulting in monocyte differentiation and fusion and thereby formation of the characteristic large giant cells [38]. These newly formed giant cells have bone resorption properties and cause the characteristic osteolysis.

The current treatment of GCTB is curettage combined with local adjuvant therapy to fill the bone cavity (e.g. polymethylmethacrylate or cancellous bone grafts) or, if necessary, en bloc resection. If tumours are unresectable or recur, patients can be treated with denosumab, a monoclonal antibody against RANK ligand [32], or bisphosphonates [39]. Neutralization of RANK ligand will inhibit the formation of giant cells and consequently the bone resorption process. However, the neoplastic stromal cells are not affected, requiring life-long treatment and causing relapse if treatment is discontinued. This underlines the need to develop novel therapeutic strategies directly targeting the neoplastic stromal cells in GCTB.

### Chondroblastoma

Chondroblastoma is a benign, cartilage-forming tumour which accounts for less than 1% of all primary bone tumours (Table 1). These tumours typically arise in skeletally immature patients ranging in age from 10 to 25 years old, predominantly in the epiphysis of long bones [18]. Chondroblastomas are successfully treated with curettage combined with bone grafting, or radiofrequency ablation. Recurrence rates are between 14 and 18%, and the development of so-called ‘benign’ pulmonary metastases is very rare. The immature cartilage cells, chondroblasts, located in growth plates are considered as the cells of origin in chondroblastoma [40], although another study suggests that these tumours deposit osteoid matrix and should be reclassified as bone-forming tumours [41]. Additionally, a variable amount of osteoclast-like giant cells is seen. Chondroblastoma differs from giant cell tumour of bone by the presence of a sclerotic rim, the relatively younger age of the patients and histological characteristics (e.g. cell type and matrix formation), although it is sometimes difficult to distinguish (Table 1).

### Histone H3.3 variants

**Fig. 1:**
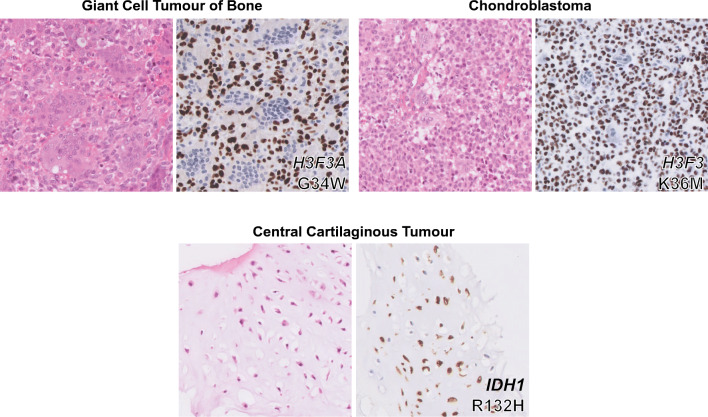
Histology and mutation-specific immunohistochemistry in chondroblastoma, giant cell tumour of bone and central cartilaginous tumours

Recently, a study was published which described the presence of histone H3.3 mutations in both chondroblastoma and GCTB [24]. Interestingly, GCTB exclusively showed alterations in the *H3F3A* gene (92% of cases), while *H3F3B* was predominantly affected in chondroblastoma (95% of cases; 93% *H3F3B* and 7% *H3F3A*). *H3F3A* and *H3F3B* are paralogous genes located on different chromosomes (chromosome 1 and chromosome 17, respectively) and have a slightly different DNA sequence, but both encode for the exact same histone H3.3 protein. The altered amino acids in these genes are remarkably specific for each tumour type. GCTB specifically harbours G34W mutations or, less common, G34L variants in *H3F3A*, while chondroblastoma exclusively shows K36M alterations in both *H3F3A* and *H3F3B* [24]. Histone H3.3 alterations have also been described in glioma and glioblastoma; especially K27M and G34R/V alterations in *H3F3A* (up to 60%) [42]. Of note, histone H3.3 variants are rarely found in other tumours, making these mutations highly specific for glioma, glioblastoma, GCTB and chondroblastoma [24, 43]. This has led to development of novel diagnostic tools that either use sequencing of *H3F3A* and *H3F3B*, or immunohistochemistry using G34W and K36M mutation-specific antibodies, to distinguish between chondroblastoma, GCTB and other giant cell-rich tumours [30, 31, 44] (Fig. 1). Their high frequency of occurrence suggests an important role for these mutations in tumourigenesis, suggesting that targeting these mutations or the underlying alterations could be used as novel therapeutic strategies. Interestingly, within the tumours, the mutation was only observed in stromal-like cells and not in cells from the osteoclast lineage, indicating that these cells are the neoplastic and driving component of GCTB and chondroblastoma [24] (Fig. 1).

### Epigenetic alterations induced by histone H3.3 variants

Histone H3.3 is a well conserved replacement histone that is highly structurally related to the canonical histone H3 protein. H3.3 is the most predominant form of histone H3 in non-dividing cells, and incorporation is independent of DNA synthesis. Usually, histone H3.3 replaces the canonical histone H3 protein in the nucleosome of active genes (e.g. promoter and gene bodies), suggesting that it may be an epigenetic regulator of transcriptionally active chromatin [45]. The most frequently modified lysine residues on histone H3.3 are K4, K9, K27 and K36, which usually acquire mono-, di- or tri-methylation and acetylation marks. Of note, two histone H3.3 mutations (i.e. K27M and K36M) are exactly at the location of these lysine residues, while the G34 mutations are closely situated near the K36 position. This implies that all H3.3 mutations could hamper the formation of modification marks and thereby change the transcription of genes across the whole genome.

Several research groups have started to elucidate the epigenetic alterations caused by H3.3 mutations and the mechanisms underlying these changes in chondroblastoma and GCTB. The K36M mutation in *H3F3A* and *H3F3B* (H3K36M) causes genome-wide reduction of methylation in chondroblastoma, specifically loss of di-methylation of H3K36 (H3K36me2) at intergenic regions and H3K36me3 at gene bodies [46, 47]. Methylation of K36 is associated with active gene transcription, indicating that the H3K36M mutation inhibits gene transcription across the whole genome [48]. Pathways that are shown to be altered are chondrogenic and osteogenic differentiation (i.e. downregulation of *BMP2* and *RUNX2*, respectively) and homologous recombination (i.e. downregulation of *BRCA1* and *ATRX*) [46].

The reduction in methylation is caused by the inhibition of histone methyltransferases (HMTs) such as NSD1, NSD2 and SETD2. The active SET domain within these HMTs contains tyrosine residues which normally bind to a lysine residue on histone tails to deposit a methylation mark. However, in the case of a H3K36M mutation, the lysine is mutated into a methionine which results in stronger binding of HMTs to the histone tail. This causes a global reduction in the HMT levels and its enzymatic activity, leading to a genome-wide alteration of the H3K36me2/3 landscape. This higher affinity of HMTs for H3K36M was confirmed by solving the crystal structure of the SETD2-H3K36M complex and by performing pull-down assays showing an enriched interaction between HMTs and H3K36M [46, 47, 49, 50].

Additionally, H3K36M mutations cause redistribution of the repressive H3K27me3 histone mark and its reader complex PRC1. Upregulation of H3K27me3 has been observed in intergenic regions, while loss was observed at gene loci [47]. Consequently, re-expression of normally H3K27me3 silenced genes occurs such as *Wnt6* and *Sox6*, genes involved in blocking mesenchymal differentiation. Thus, H3K36M mutations change the K27 and K36 methylation landscape, resulting in a highly altered transcriptome.

In contrast, the mechanism underlying the observed epigenetic changes in cells harbouring G34 mutations in *H3F3A* (H3F3A G34) is less clear. Although histone modifications cannot be directly made on the G34 residue, H3F3A G34 mutations may affect the deposition of marks on the K36 position, and possibly also other modification sites, due to steric hindrance. An interaction study showed that the G34 position is involved in binding SETD2 [50]. SETD2 has a narrow binding pocket at the G34 position which can only bind the smallest amino acid: glycine. Consequently, all H3F3A G34 mutations will prevent SETD2 from binding histone H3.3, leading to a decrease in H3K36me3. However, a genome-wide reduction of H3K36me2/3 was not observed in cells harbouring H3F3A G34R/V mutations, but only at sites where the mutated histone was localized [51]. The inhibitory effect on the enzymatic activity of SETD2 has also been confirmed for H3F3A G34W/L mutations in GCTB, with a concomitant decrease in H3K36me3 and, noticeably, an increase in the repressive H3K27me3 mark on the mutated histone tails [52]. However, another study found an increase of the H3K36me3 mark in GCTB harbouring H3F3A G34W/V mutations [44]. Moreover, in glioblastoma, H3F3A G34V mutations inhibit the enzymatic activity of KDM4 H3K36me3 demethylases, leading to increased H3K36me3 and H3K9me3 in histone H3.3 enriched regions [53]. This suggests that H3F3A G34 mutations can both increase and decrease the H3K36me3 mark at particular sites in the genome, leading to significant change in the transcriptome of cells. In high-grade H3F3A G34V-mutated glioma, this leads to a change in the methylation pattern of approximately 150 genes (e.g. the oncogene *MYCN*) [54].

For GCTB, the genes undergoing a change upon redistribution of the H3K36me3 marks as a result of the H3F3A G34W/L mutations remain to be identified. Changes that are observed in primary GCTB cells harbouring a G34W mutation are increased proliferation, migration and colony formation capacity as compared to wild-type counterparts [55, 56]. Furthermore, splicing aberrations and alternative transcription start sites are frequently observed in H3F3A G34W-mutated cells, suggesting an alteration in the RNA processing pathway [55]. Future studies are needed to elucidate which genes and signalling pathways are affected by the H3F3A G34 mutation-driven epigenetic alterations.

### Therapeutic implications for epigenetic alterations induced by histone H3.3 variants

Currently, there are limited reports on in vitro or in vivo studies describing promising epigenetic therapeutic strategies for GCTB. Therapies directly targeting the mutated histones are lacking, and reversal of genome-wide epigenetic alterations is not feasible without major side-effects on the healthy, normal epigenetic landscape. However, the use of histone demethylase inhibitors, such as GSKJ4, may counteract the observed reduction or redistribution of H3K36me3 and H3K27me3. On the other hand, reduction of H3K36me3 may be a promising therapeutic target and recently it was shown that WEE1 inhibition in GCTB results in a global reduction of H3K36me3 and a decrease in proliferation [35]. Nevertheless, further research is needed to identify the genes affected by the epigenetic changes to develop targeted, effective therapeutic strategies. Additionally, most research has been performed on methylation changes across the genome, while the effect on other modification marks, such as acetylation, phosphorylation and ubiquitination, has not been examined yet. Another promising therapeutic strategy could be to stimulate the enzymatic activity of SETD2 and KDM4 or to overcome the strong binding between KDM4-H3F3A G34, but this kind of compounds are currently lacking.

## *IDH1* and *IDH2* mutations in Enchondroma and Chondrosarcoma

### Enchondroma and Chondrosarcoma

Enchondroma is a benign, cartilage producing tumour which accounts for 10–25% of all benign bone tumours [19] (Table 1). These tumours can occur at all ages and mainly affect bones of the hands and feet and long tubular bones. Normally, enchondromas are not treated, since most tumours are solitary and rarely undergo malignant transformation. Non-hereditary syndromes causing multiple enchondromas (i.e. Ollier disease and Maffucci syndrome) increase the risk of progression to chondrosarcoma to ~ 40% [57].

Chondrosarcomas are malignant, cartilage-forming tumours and make up 20% of all malignant bone tumours [20] (Table 1). These tumours usually develop during adulthood, mainly affecting the pelvis and long bones, and either arise as primary tumours (i.e. in a previously healthy bone) or as secondary tumours (i.e. in a benign precursor lesion). Conventional chondrosarcoma is the most prevalent form (85%), followed by dedifferentiated chondrosarcoma (10%). Most conventional chondrosarcomas arise in the medulla of the bone (central conventional chondrosarcoma). Histological grading is considered as the most important prognostic marker to predict the 10-year survival rate of conventional chondrosarcoma patients: 88% for atypical cartilaginous tumours and chondrosarcoma grade I (ACT/Grade I), 62% for Grade II and 26% for Grade III [23]. Furthermore, the metastatic potential of conventional chondrosarcoma also increases in higher grade tumours: 0%, 10% and 71% for ACT/Grade I, Grade II and Grade III, respectively [21]. Chondrosarcomas are intrinsically resistant towards conventional chemo- and radiotherapy, and surgery is considered as the only curative treatment option. Although mutations in for instance *IDH1* and *-2, COL2A1* and *TP53* combined with aberrations in signalling pathways such as IHH/PTHrP, pRB and PI3K/mTOR have been identified as key molecular changes in chondrosarcoma [20], the development of novel targeted therapies has not yet been successful.

### *IDH1* and *IDH2* mutations

Eighty-seven percent of the solitary and multiple enchondromas, ~ 50% of the primary central conventional chondrosarcomas and 86% of the secondary central chondrosarcomas harbour heterozygous point mutations in *IDH1* and *IDH2* [6, 25, 26]. The introduction of an *IDH1* mutation alone is sufficient to induce enchondromatosis in mice [58]. Furthermore, introduction or imitation of *IDH* mutations in mesenchymal stem cells impairs osteogenic differentiation and promotes chondrogenic differentiation in vitro [59, 60]. Thus, *IDH1* and *IDH2* mutations represent an early event in the development of central cartilaginous tumours. Over time, central chondrosarcomas acquire additional mutations (e.g. *COL2A1* and *TP53*), which probably become more important drivers of tumourigenesis than *IDH* mutations [61].

*IDH1* and *IDH2* mutations also frequently occur in for instance acute myeloid leukaemia (AML), glioma and cholangiocarcinoma [62]. Most of the point mutations occur at specific arginine residues: the R132 position in *IDH1* and the R140/R172 positions in *IDH2.* Some of these variants can be detected with the use of immunohistochemistry and mutation-specific antibodies, displaying the mosaicism for *IDH* mutations in central cartilaginous tumours (Fig. 1). The identification of *IDH* mutations in central cartilaginous tumours facilitates the diagnosis of dedifferentiated chondrosarcoma on a small biopsy, and the distinction between chondroblastic osteosarcoma and central chondrosarcoma [63]. Interestingly, the most frequent point mutations in either *IDH1* or *IDH2* differ among the above listed tumour types. Central chondrosarcoma and cholangiocarcinoma predominantly harbour R132C mutations in *IDH1* (50%), glioma has mainly R132H mutations in *IDH1* (90%) and AML has most often R140Q mutations in *IDH2* (30–50%) [62]. However, none of the mutations are exclusively observed in one tumour type, suggesting a similar effect of all *IDH1* and *IDH2* mutations on tumourigenesis.

IDH1 and IDH2 are enzymes with a similar function in the tricarboxylic acid cycle (TCA cycle) where they convert isocitrate into α-ketoglutarate (α-KG) and CO_2_. Due to the arginine point mutations, the IDH enzymes acquire a gain-of-function, leading to additional conversion of α-KG into the oncometabolite D-2-hydroxyglutarate (D-2-HG). Of note, different *IDH1* and *IDH2* mutations produce variable levels of D-2-HG: R132C is a strong D-2-HG producer, while R132H and R140Q produce lower levels of the oncometabolite [62]. This could explain why some tumour types harbour specific point mutations more frequently than others, suggesting that enchondromas rely on high levels of the oncometabolite.

D-2-HG and α-KG have a high structural similarity, leading to competitive binding of α-KG-dependent enzymes by D-2-HG. Some of these α-KG-dependent enzymes are involved in maintaining the epigenetic landscape of cells, such as DNA demethylases (family of TET enzymes) and histone demethylases (family of Jumonji enzymes). *IDH* mutations also affect other processes within the cell, such as metabolism, cell growth signalling pathways and DNA damage repair [64], but these alterations go beyond the scope of this review.

### Epigenetic alterations induced by *IDH1* and *IDH2* mutations

It has been shown that D-2-HG inhibits the activity of Tet Methylcytosine Dioxygenase 2 (TET2) in vitro [65]. Normally, TET2 mediates the demethylation of DNA by hydroxylation of 5-methylcytosine (5-mC) to 5-hydroxymethylcytosine (5-hmC), which results in a DNA hypermethylation phenotype if this enzyme is inhibited by D-2-HG. Several studies confirmed the global DNA hypermethylation phenotype in several *IDH*-mutated tumours, including enchondroma and central chondrosarcoma [6, 66]. However, an immunohistochemistry study including 9 enchondromas and 92 central chondrosarcomas showed that the 5-mC and 5-hmC levels are highly variable and not associated with the *IDH* mutation status [67]. Additionally, long-term treatment of chondrosarcoma cell lines with the *IDH1* mutant inhibitor AGI-5198 does not alter the DNA methylation phenotype, suggesting that the epigenetic alterations might have become independent of the *IDH1* mutation [61]. Another study also observed a persistence of the aberrant DNA methylation phenotype in 25% of the loci after withdrawal of doxycycline induced expression of the *IDH1* mutant in astrocytes [68]. To conclude, enchondromas and central chondrosarcomas have an altered DNA methylation phenotype, but this might have become partially static and no longer directly dependent on D-2-HG mediated inhibition of TET2.

Another group of enzymes that are inhibited by D-2-HG is the Jumonji-C domain-containing histone lysine demethylases (KDM enzymes), including the KDM4 family members which are also affected by histone H3.3 mutations [69]. Inhibition of these enzymes results in a global increase of several di- and tri-methylation marks on histone tails. Introduction of an *IDH1* or *IDH2* mutation in HEK293T cells leads to elevated levels of the H3K4me3, H3K9me2/3, H3K27me3, H3K36me3 and H3K79me2 histone marks [70]. An induction in H3K9me3 and H3K27me3 is also observed in glioma samples harbouring an endogenous *IDH1* mutation [70]. However, in vivo studies with *IDH1* mutant knock-in mice show that none or only a subset (i.e. H3K4me3, H3K36me3 and H3K79me3) of the histone methylation marks are aberrant [70, 71]. This implies that an *IDH* mutation alone is not enough to alter the histone methylation landscape. Similar conflicting results are also observed in enchondroma and central chondrosarcoma: an immunohistochemistry study on 101 primary tumours shows high levels of the H3K4me3, H3K9me3 and H3K27me3 histone modification marks, irrespective of the *IDH1* or *IDH2* mutation status [67]. Additionally, long-term AGI-5198 treatment of chondrosarcoma cell lines could not alter the expression of H3K4me3, H3K9me3 and H3K27me3 [61]. Both studies suggest that the methylation histone modification marks in enchondromas and central chondrosarcoma are regulated by additional mechanisms besides D-2-HG-dependent inhibition of the KDM enzymes.

Although the specific genes affected by *IDH* mutation-induced epigenetic alterations remain to be elucidated, most studies point towards reduced differentiation capacity. Both neural and haematopoietic differentiations are impaired due to aberrant histone methylation marks or DNA hypermethylation [70, 72]. In *IDH1*-mutated cholangiocarcinoma, reduced expression of H3K4me3 at the *HNF4A* promoter inhibits hepatocyte differentiation [73]. Furthermore, introduction of an *IDH* mutation or addition of D-2-HG impairs osteogenic differentiation and induces chondrogenic differentiation in mesenchymal stem cells [59, 60]. *IDH* mutations also reduce the expression of DNA repair protein ATM [74], although these findings have not yet been described in central chondrosarcoma and are contradictory to the observed chemo- and radiotherapy resistance in these tumours. Furthermore, hypermethylation of the *NAPRT1* promoter seems to correlate to NAMPT inhibitor sensitivity in *IDH*-mutated glioma [75]. Chondrosarcoma cell lines are also sensitive to NAMPT inhibition and show a hypermethylated *NAPRT1* promoter, although this phenotype is independent of the *IDH* mutation status [76].

### Therapeutic implications for epigenetic alterations induced by *IDH1* and *IDH2* mutations

The most straightforward way to target the epigenetic alterations is direct inhibition of the *IDH1* or *IDH2* mutant enzyme and thereby prevention of the formation of the oncometabolite. However, in vitro experiments do not show a beneficial effect in chondrosarcoma cell lines: proliferation, migration and colony formation capacity are not affected, only at high concentrations [61, 77]. Several clinical trials are ongoing to evaluate the effect of *IDH* mutant inhibitors in patients, but the results for chondrosarcoma patients have not yet been published. Since several in vitro studies indicate that the observed changes might have become static and independent of the *IDH* mutation over time, it may be more promising to directly target these changes. To counteract the DNA hypermethylation phenotype, the use of DNA methyltransferase inhibitors, such as decitabine, could be a promising therapeutic strategy. An in vitro study shows that decitabine induces the re-expression of several epigenetically silenced tumour suppressor genes and reduces the proliferation and migration of chondrosarcoma cells [78]. Contradictory to these findings, use of decitabine in the Swarm rat chondrosarcoma model results in a more progressive phenotype [79]. These conflicting results indicate that more research is needed before epigenetic therapies can be used for central chondrosarcoma patients. For example, identification of the genes that are mainly affected by the epigenetic alterations could lead to more targeted therapeutic approaches instead of global DNA demethylation. Besides that, the non-epigenetic effects of D-2-HG could also play a major role in tumourigenesis, warranting a more wide-spread therapeutic approach to target multiple D-2-HG-affected pathways at once.

## Conclusion and future perspectives

**Fig. 2:**
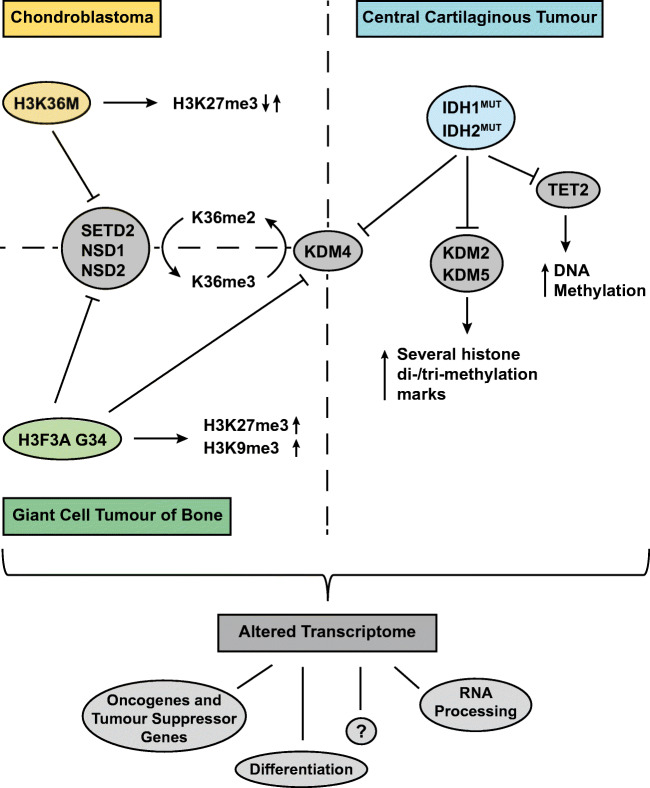
Schematic overview of the individual and shared epigenetic alterations in chondroblastoma, giant cell tumour of bone and central cartilaginous tumours

Chondroblastoma, GCTB and central cartilaginous tumours all harbour driver mutations that induce wide-spread epigenetic alterations (Fig. 2). Interestingly, these epigenetic alterations do not seem to be associated with malignant potential, as the *IDH* and H3.3 mutations are considered drivers only in benign (enchondroma, chondroblastoma) and locally aggressive (GCTB) neoplasms. This is in line with the fact that these epigenetic changes predominantly affect differentiation, instead of proliferation. In the case of malignant chondrosarcoma, the *IDH* mutation is no longer associated with tumourigenic behaviour and probably other mutations have taken over the driver function of the *IDH* mutation [61]. Similarly, metastases in GCTB are associated with downregulation of decorin and lumican [80]. Further research is needed to identify the genes affected by the mutation-driven epigenetic alterations in the different tumour types, as these genes probably differ between the tumour types. Moreover, it remains to be elucidated why the occurrence of certain histone H3.3 mutations is highly tumour type specific. This will hopefully lead to a better understanding of the biological mechanisms underlying the development of these bone neoplasms.

The identification of specific histone H3.3 variants in both chondroblastoma (i.e. H3K36M) and GCTB (i.e. H3F3A G34) has led to development of novel diagnostic tools. Mutant protein-specific immunohistochemistry and/or sequencing of *H3F3A* and *H3F3B* both support pathologists to differentiate between chondroblastoma, GCTB and other giant cell containing neoplasms [30, 31, 44] (Table 1). While the *IDH1* R132H mutation-specific antibody is commonly used in neuropathology [81], an antibody recognizing the most common *IDH1* R132C mutation in central cartilaginous tumours is not yet available but would be very helpful in distinguishing enchondroma and chondrosarcoma from their histologic mimics [63] (Table 1).

Of note, only the neoplastic stromal-like cells harbour the histone H3.3. variants, both in chondroblastoma and GCTB, which suggests a close interplay between wild-type (e.g. giant cells) and mutant cells. A similar relation between wild-type and mutant cells has been observed for central cartilaginous tumours, which display intra-neoplastic mosaicism for the *IDH* mutations. This makes it tempting to speculate that this close interplay between wild-type and mutant cells is essential for the development of these bone neoplasms driven by epigenetic alterations.

As only ~ 50% of the chondrosarcomas harbour *IDH1* or *IDH2* mutations, alternative driving mechanisms should be considered. It remains to be elucidated whether these alternative driver mechanisms induce similar epigenetic alterations as observed in *IDH* mutant chondrosarcomas. Also, one could speculate that the *IDH* wild-type cells may overgrow the *IDH* mutant cell population over time. While *IDH* mutations are essential for the initiation of enchondroma, the additional acquired mutations that take over the driver function of *IDH* mutations in chondrosarcoma may occur in the wild-type cells which might explain the observed *IDH* wild-type genotype in half of the chondrosarcomas.
